# Temporal pattern of expression and colocalization of microglia/macrophage phenotype markers following brain ischemic injury in mice

**DOI:** 10.1186/1742-2094-8-174

**Published:** 2011-12-10

**Authors:** Carlo Perego, Stefano Fumagalli, Maria-Grazia De Simoni

**Affiliations:** 1 Laboratory of Inflammation and Nervous System Diseases, Department of Neuroscience, Mario Negri Institute for Pharmacological Research, Via La Masa, 19-20156 Milan, Italy

**Keywords:** Inflammation, stroke, alternative activation

## Abstract

**Background:**

Emerging evidence indicates that, similarly to what happens for peripheral macrophages, microglia can express different phenotypes depending on microenvironmental signals. In spite of the large literature on inflammation after ischemia, information on M/M phenotype marker expression, their colocalization and temporal evolution in the injured brain is lacking. The present study investigates the presence of microglia/macrophage phenotype markers, their temporal expression, whether they are concomitantly expressed by the same subpopulation, or they are expressed at distinct phases or locations in relation to the ischemic lesion.

**Methods:**

Volume of ischemic lesion, neuronal counts and TUNEL staining were assessed in C57Bl/6 mice at 6-12-24-48 h and 7d after permanent occlusion of the middle cerebral artery. At the same time points, the expression, distribution in the lesioned area, association with a definite morphology and coexpression of the microglia/macrophage markers CD11b, CD45, CD68, Ym1, CD206 were assessed by immunostaining and confocal microscopy.

**Results:**

The results show that: 1) the ischemic lesion induces the expression of selected microglia/macrophage markers that develop over time, each with a specific pattern; 2) each marker has a given localization in the lesioned area with no apparent changes during time, with the exception of CD68 that is confined in the border zone of the lesion at early times but it greatly increases and invades the ischemic core at 7d; 3) while CD68 is expressed in both ramified and globular CD11b cells, Ym1 and CD206 are exclusively expressed by globular CD11b cells.

**Conclusions:**

These data show that the ischemic lesion is accompanied by activation of specific microglia/macrophage phenotype that presents distinctive spatial and temporal features. These different states of microglia/macrophages reflect the complexity of these cells and their ability to differentiate towards a multitude of phenotypes depending on the surrounding micro-environmental signals that can change over time. The data presented in this study provide a basis for understanding this complex response and for developing strategies resulting in promotion of a protective inflammatory phenotype.

## Background

Microglia, the major cellular contributors to post-injury inflammation, have the potential to act as markers of disease onset and progression and to contribute to neurological outcome of acute brain injury. They are normally present in the healthy brain where they actively survey their surrounding parenchyma by protracting and retracting their processes and they are endowed with the capacity to rapidly respond to injury or alterations in their microenvironment [1-3]. After acute brain injury, these resident cells are rapidly activated and undergo dramatic morphological and phenotypic changes. Typical morphological changes associated with microglia activation include thickening of ramifications and of cell bodies followed by acquisition of a rounded amoeboid shape. This intrinsic response is associated to recruitment of blood-born macrophages which migrate into the injured brain parenchyma [4,5]. This process is accompanied by expression of novel surface antigens and production of mediators that build up and maintain the inflammatory response of the brain tissue. Activated microglia and recruited macrophages (which are antigenically not distinguishable, henceforth referred to as M/M), can affect neuronal function and promote neurotoxicity through the release of several harmful components such as IL-1β, TNF-α, proteases and reactive oxygen and nitrogen species [6,7]. On the other hand they also possess protective qualities and promote neurogenesis and lesion repair [8-10]. Indeed, microglia have been proposed to be beneficial by several mechanisms including glutamate uptake [11] removal of cell debris [12] and production of neurotrophic factors such as IGF-1 [13], GDNF [14] and BDNF [15,16].

Studies addressing phenotypic changes occurring in macrophages in peripheral inflammation and immunity have shown that these cells can undergo different forms of polarized activation. One is the classic or M1 activation, characterized by high capacity to present antigen, high production of NO and ROS and of proinflammatory cytokines. M1 cells act as potent effectors that kill micro-organisms and tumor cells, drive the inflammatory response and may mediate detrimental effects on neural cells. The second phenotype (M2) is an alternative apparently beneficial activation state, more related to a fine tuning of inflammation, scavaging of debris, promotion of angiogenesis, tissue remodeling and repair. Specific environmental signals are able to induce these different polarization states [17]. A similar possibility has been also recently raised for microglia, by showing that these cells, under certain conditions, can indeed be pushed to both extremes of the M1 and M2 differentiation spectrum [16,18]. More studies are needed to substantiate these observations.

In this frame the present study aims at getting insight on previously unexplored aspects of microglia phenotype changes induced by cerebral ischemia, namely, the presence of specific phenotype markers, their temporal expression, whether or not they are concomitantly expressed by the same subpopulation, whether they are expressed at distinct phases or locations in relation to the ischemic lesion. We focussed on a few molecules that are known to be expressed by macrophages in peripheral inflammation and that have been associated to different functions. They include: CD11b, a marker of M/M activation/recruitment, CD45 expressed on all nucleated hematopoietic cells [19], CD68 a marker of active phagocytosis, Ym1 a secretory protein that binds heparin and heparin sulphate and CD206 a C-type lectin carbohydrate binding protein, both of them expressed by alternatively activated macrophages and associated to recovery and function restoration [20,21].

## Methods

### Animals

Male C57Bl/6 mice (10-week old, 20-25 g, Harlan Laboratories, Italy) were used. Procedures involving animals and their care are conducted in conformity with the institutional guidelines (Quality Management System Certificate - UNI EN ISO 9001:2008 - Reg. N° 8576-A) that are in compliance with national (D. Lvo. n. 116, 27 Gennaio 1992; Legge n° 413, 12 Ottobre 1993; Circolare No. 8, 22 Aprile 1994; D.M. 29/09/95; D.M. 26/04/2000) and international (EEC Council Directive 86/609, OJ L 358, 1, Dec. 12, 1987; Guide for the Care and Use of Laboratory Animals, U.S. National Research Council, 1996) laws and policies. Before beginning any procedure, mice were housed for at least 1 week in their home cages at a constant temperature, with a 12 hour light-dark cycle, and *ad libitum *access to food and water in a selective pathogen-free (SPF) *vivarium*.

### Focal cerebral ischemia

Permanent ischemia was obtained by permanent middle cerebral artery occlusion (pMCAO) [22,23]. Briefly, mice were anesthetized with Equitensin (pentobarbital 39 mM, chloral hydrate 256 mM, MgSO4 86 mM, ethanol 10% v/v, propyleneglycol 39.6% v/v) 100 μl/mouse administered by intraperitoneal (i.p.) injection. A vertical midline incision was made between the right orbit and tragus. The temporal muscle was excised, and the right MCA was exposed through a small burr hole in the left temporal bone. The *dura mater *was cut with a fine needle, and the MCA permanently occluded by electrocoagulation just proximal to the origin of the olfactory branch. Intraoperative rectal temperature was kept at 37.0 ± 0.5°C using a heating pad (LSI Letica). Mortality rate was 8.5%. Sham-operated mice received identical anesthesia and surgical procedure without artery occlusion.

### Experimental design and blinding

Mice were assigned to surgery and experimental groups with surgery distributed equally across cages and days. To minimize the variability, all surgeries were performed by the same investigator, blinded to the experimental groups. All subsequent histological and immunohistological evaluations were also done by blinded investigators.

### Brain transcardial perfusion

At selected time points mice were deeply anesthetized with Equitensin (120 μl/mouse i.p.) and transcardially perfused with 20 ml of PBS, 0.1 mol/liter, pH 7.4, followed by 50 ml of chilled paraformaldehyde (4%) in PBS. After carefully removing the brains from the skull, they were transferred to 30% sucrose in PBS at 4°C overnight for cryoprotection. The brains were then rapidly frozen by immersion in isopentane at - 45°C for 3 min before being sealed into vials and stored at -70°C until use.

### Quantification of infarct size

For lesion size determination, 20-μm coronal brain cryosections were cut serially at 320-μm intervals and stained with Cresyl Violet [8]. On each slice, the infarcted area was assessed blindly and delineated by the relative paleness of histological staining tracing the area on a video screen. The infarcted area and the percentage of brain swelling for edema correction were determined by subtracting the area of the healthy tissue in the ipsilateral hemisphere from the area of the contralateral hemisphere on each section [24,25]. Infarct volumes were calculated by the integration of infarcted areas on each brain slice as quantified with computer- assisted image analyzer and calculated by Analytical Image System (Imaging Research Inc., Brock University, St. Catharines, Ontario, Canada).

### Slice selection and quantitative analysis

**Figure 1:**
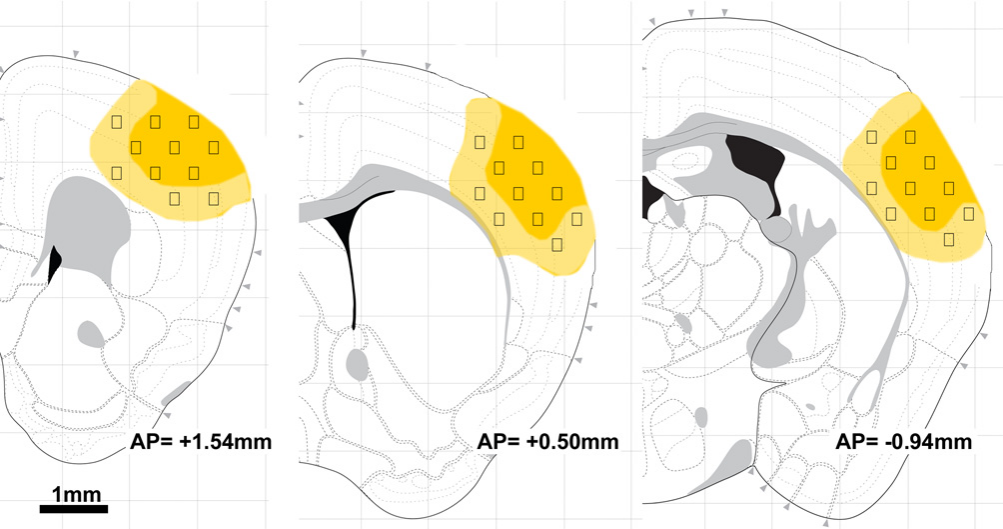
**Slices selection and tissue sampling for neuronal counts and quantification of immunostained area**. Fields for neuronal counts, TUNEL evaluation and quantification of stained area were positioned within the ischemic territory at defined distances (see methods for details). The regions sampled pertained to the ischemic territory (yellow) at the time points considered (6 h, 12 h, 24 h, 48 h and 7d).

Three brain coronal sections per mouse (+1.54, +0.50 and - 0.94 mm from bregma, KBJ Franklin and G Paxinos, The Mouse Brain in Stereotaxic Coordinates, Academic Press), were used to quantify the stained area. On each slice, anatomically defined cortical regions of interest were demarcated, corresponding to the primary motor cortex, somatosensory cortex, insular cortex (granular, agranular) and secondary somatosensory cortex, representing the cortical regions involved in the largest lesion extension observed at 24 h after ischemia. Field selection was performed using a BX61 Olympus microscope equipped with a motorized stage acquiring the same focal plan throughout the samples [26]. For neuronal count, CD11b and CD68 quantification fields at 40× magnification were selected at 1.54 mm anterior to bregma (11 fields), at 0.50 mm anterior to bregma (11 fields) and at 0.94 mm posterior to bregma (11 fields). The first raw of fields was positioned at the lesion edge, spacing each field by 572.5 μm (distance between centres of the fields). Further raws of fields were positioned distanced by 389.3 μm each. For TUNEL, CD45, Ym1 and CD206 twenty-four quantification fields at 20× magnification were selected. Eight fields per slice were selected and fields were separated by 572.5 μm (distance between centres of fields), while distance between each raw was 389.3 μm. A schematic representation of the regions of interest and of the selected fields is depicted in Figure 1.

### Neuronal Count

Cresyl Violet stained brain sections were used for neuronal count. Thirty-three fields at 40× were analyzed for each mouse. The amount of neuronal loss was calculated by pooling the number of stained neurons in the three ipsilateral sections and expressed as percentage of those in sham- operated animals. Fields were analyzed using ImageJ software http://rsbweb.nih.gov/ij/ and segmentation was used to discriminate neurons from glia on the basis of cell size.

### TUNEL staining

To assess the presence of injured cells showing DNA damage, terminal deoxynucleotidyl transferaseYmediated dUTP nick end labeling (TUNEL) staining was performed on 20-μm sections by *in situ *cell death detection kit (Roche, Mannheim, Germany) according to the manufacturer instructions, as previously described [27]. DNase-treated sections were used as a positive control. After staining, the sections were visualized using fluorescent microscopy (Olympus IX70 Olympus Tokyo, Japan). Images of the area of interest were acquired using AnalySIS software (Olympus, Tokyo, Japan). For each mouse twenty-four fields at 20× were analyzed. TUNEL-positive cells were counted using ImageJ software http://rsbweb.nih.gov/ij/ and expressed as number per mm^2 ^for subsequent statistical analysis [28].

### Immunohistochemistry

Immunohistochemistry was performed on 20-μm brain coronal sections using anti-mouse CD11b (1:700, kindly provided by Dr. Doni, [8] anti-mouse-CD45 (1:800, BD Biosciences Pharmigen, San Jose, CA), anti-mouse CD68 (1:200, Serotec, Kidlington, UK), anti-mouse Ym1 (1:400, Stem Cell Technologies, Vancouver, Canada), anti-mouse CD206 (1:100, Serotec, Kidlington, UK). Positive cells were stained by reaction with 3, 3 diaminobenzidine tetrahydrochloride (DAB, Vector laboratories, CA, USA). For negative control staining, the primary antibodies were omitted and no staining was observed. CD45-positive cells displayed 2 morphologies: a leukocyte-like shape corresponding to cells with a rounded cell body without branches and high expression of CD45 (CD45^high^), and a microglia-like shape having a small cell body and several branches and a fainter expression of CD45 (CD45^low^) [22]. Quantification was carried out on CD45^high ^cells. Immunostained area for each marker was measured using ImageJ software http://rsbweb.nih.gov/ij/ and expressed as positive pixels/total assessed pixels and indicated as staining percentage area (as number per mm^2 ^for CD206) for subsequent statistical analysis [28].

### Immunofluorescence and confocal analysis

Immunofluorescence was performed on 20-μm coronal sections according to the previously described method [22]. Primary antibodies used were: anti-mouse CD45 (1:800 or 1:1500); anti-mouse Ym1 (1:400, Stem Cell Technologies, Vancouver, Canada), anti-mouse CD206 (1:100, Serotec, Kidlington, UK), anti-mouse CD11b (1:30000, kindly provided by Dr. Doni), anti-mouse CD68 (1:200, Serotec, Kidlington, UK), anti-mouse NeuN (1:250, Millipore, Billerica, MA, USA). Fluorconjugated secondary antibodies used were: Alexa 546 anti-rat, Alexa 594 anti-rabbit, Alexa 488 anti-mouse (all 1:500, Invitrogen, Carlsbad, CA). Biotinilated anti-rat antibodies (1:200, Vector Laboratories, Burlingame, CA) were also used followed by fluorescent signal coupling with streptavidine TSA amplification kit (cyanine 5, Perkin Elmer, MA, USA). Similarly to what reported for immunohistochemistry DAB staining, also in this case we considered only cell displaying CD45 rounded morphology (CD45^high^, [22]). Appropriate negative controls without the primary antibodies were performed. None of the immunofluorescence reactions revealed unspecific fluorescent signal in the negative controls. Immunofluorescence was acquired using a scanning sequential mode to avoid bleed-through effects by an IX81 microscope equipped with a confocal scan unit FV500 with 3 laser lines: Ar-Kr (488 nm), He-Ne red (646 nm), and He-Ne green (532 nm) (Olympus, Tokyo, Japan) and a UV diode.

Two main areas of interest were considered, namely ischemic core and border zone [29] at both 24 h and 7d after pMCAo Three-dimensional images were acquired over a 10 μm z-axis with a 0.23 μm step size and processed using Imaris software (Bitplane, Zurich, Switzerland) and Photoshop cs2 (Adobe Systems Europe Ltd).

### Statistical analysis

Statistical power (1-β) was assessed as post-hoc analysis by means of G*Power [30]. Statistical analysis was performed using standard software packages GraphPad Prism (GraphPad Software Inc., San Diego, CA, USA, version 4.0). All data are presented as mean and standard deviation (sd). The comparison between groups was performed using One-way ANOVA followed by appropriate *post hoc *test. p-values lower than 0.05 were considered statistically significant.

## Results

### Histopathological findings at different time points from pMCAO

**Figure 2:**
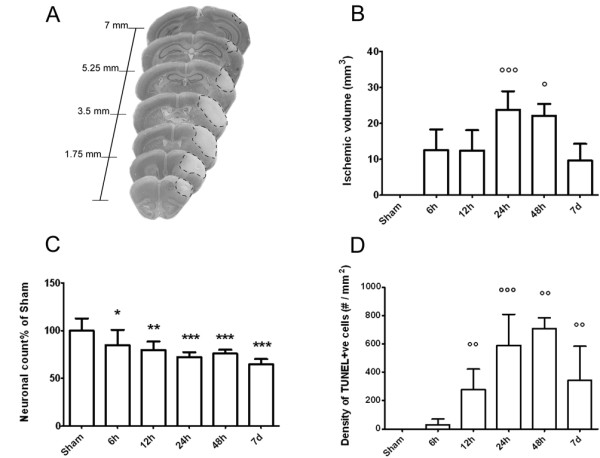
**Histopathological findings at different time points from pMCAO**. A: representative brain coronal sections obtained 24 h after pMCAO. Pale demarcated areas depict the ischemic lesion. Quantification of ischemic volume (B), neuronal counts (data obtained from the mean of 33 frames/mouse and expressed as % of sham groups, C) and TUNEL-positive cells (D) in the cortex of sham and ischemic mice at different times after pMCAO. Data are reported as mean+sd, n = 8. *p < 0.05, **p < 0.01, ***p < 0.001 versus sham; °p < 0.05, °°p < 0.01, °°°p < 0.001 versus 6 h, Bonferroni's Multiple Comparison Test.

pMCAO induced an infarcted area in the ipsilateral cortex (Figure 2A) as expected [22,23]. The lesion, evaluated as relative paleness of cresyl violet staining and corrected for edema, at 6 h, 12 h, 24 h, 48 h and 7d, had a volume of 12.5 mm^3 ^± 5.8, 12.4 mm^3 ^± 5.7, 23.8 mm^3 ^± 5.1, 22.1 mm^3 ^± 3.3 and 9.6 mm^3 ^± 4.7, respectively (Figure 2B).

Cortex, the brain area involved in the ischemic lesion was considered for neuronal count (Figure 2C). Six hours after ischemia, neuronal count performed in the ipsilateral cortex revealed a significant cell loss when compared to the corresponding area in the sham-operated group (84.9%). Neuronal counts progressively but slowly decreased reaching 64.9% at 7d. No significant difference was found between ispilateral and contralateral side in sham-operated animals (data not shown).

At 6 h after pMCAO rare TUNEL-positive cells were present in the injuried cortex indicating the presence of few dying cells (30.2 ± 14.2, expressed as cell density per mm^2^, Figure 2D). Number of dying cells progressively increased at 12, 24 and 48 h post ischemia (278.6 ± 51.1, 589.7 ± 77.3 and 708.8 ± 30.2, respectively). Seven days after ischemia still several TUNEL-positive cells were present (343.6 ± 120.0) indicating the persistence of dying cells at this time point. Positive TUNEL staining was not apparent in any sham-operated mice at any time points.

### Time-course of expression of M/M markers: CD11b, CD45, CD68, Ym1, CD206

The M/M markers expression was analyzed within the ischemic area based on the tissue sampling represented in Figure 1. At each time point, the sampled cortical area pertained to the ischemic territory, being the number of neurons in this region decreased compared to sham animals at every time points (Figure 2C).

**Figure 3:**
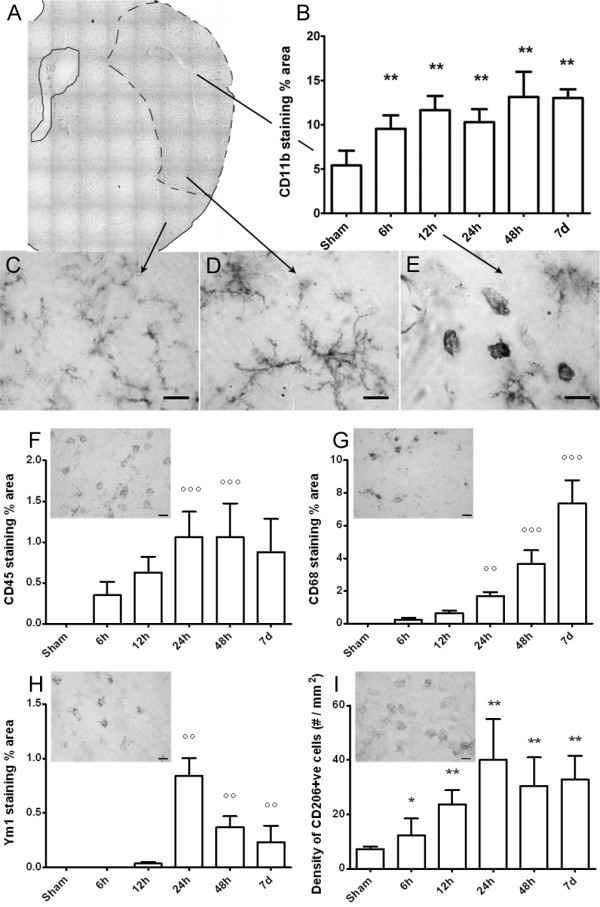
**Immunohistochemical analysis and quantification of microglial markers: CD11b, CD45, CD68, Ym1, CD206**. A: representative micrographs of CD11b immunostaining in the injured cortex at 24 hours after pMCAo. CD11b-positive cells display different morphology depending on their localization in the ischemic area (C: outside the lesion, D: border zone, E: ischemic core). B: quantification of CD11b immunostaining at different times after pMCAo. Representative micrographs of CD45 (F), CD68 (G), Ym1 (H), CD206 (I) immunoreactivity at 24 hours after ischemia, and related quantifications at different times after pMCAO (Bar 10 μm). Data are expressed as mean+sd of 33 frames/mouse (24 frames/mouse for CD45, TUNEL, CD206), n = 8. One way Anova: p < 0.0001. *p < 0.05, **p < 0.01 vs sham; °°p < 0.01, °°°p < 0.001 vs 6 h (12 h for Ym1). Bonferroni's Multiple Comparison Test.

CD11b, a constitutive marker of microglia and macrophages was expressed at every time point considered as well as in sham-operated mice (5.6 ± 1.9, percent of stained area). Starting from 6 h the immunoreactivity increased and remained elevated at every subsequent time point considered, with no major differences throughout the experimental groups (9.5 ± 1.5, 11.7 ± 1.6, 10.1 ± 1.7, 13.1 ± 2.8, 13.0 ± 0.1, respectively at 6 h, 12 h, 24 h, 48 h and 7d, Figure 3B).

Outside the lesion, CD11b staining revealed thin ramifications and small soma (Figure 3A/C). CD11b immunoreactivity was associated with a different morphology in relation to the cell localization in the lesioned area. Two main areas were identified, namely a lesion border showing CD11b+ highly ramified cells (Figure 3A/D) and an ischemic core showing both CD11b+ ameboid cells and cells with hypertrophic soma endowed with thick branches (Figure 3A/E).

No CD45-positive cells (CD45^high ^cells, see methods, Figure 3F) could be observed in sham-operated mice and in the contralateral hemisphere of ischemic mice. Six hours after ischemia these cells were clearly visible in the area considered (0.4 ± 0.2 percent of stained area). The immunoreactivity was further increased 12 and 24 h after ischemia (0.6 ± 0.2 and 1.1 ± 0.3, respectively). No further increase could be observed at 48 h (1.1 ± 0.4). CD45 staining was still present at 7d (0.9 ± 0.4, Figure 3F).

CD68 immunoreactivity was undetectable in sham-operated mice and appeared 6 h after ischemia (0.3 ± 0.2 percent of stained area). It progressively increased at every time point considered (0.6 ± 0.2 at 12 h; 1.7 ± 0.2 at 24 h; 3.7 ± 0.8 at 48 h). Notably, a dramatic increase in the CD68 stained area could be observed at 7d (7.4 ± 1.4, Figure 3G).

Ym1 immunoreactivity was detectable starting from 12 h (0.04 ± 0.02 percent of stained area). This marker was maximally expressed at 24 h (0.84 ± 0.16) and markedly decreased at later time points (0.37 ± 0.10 at 48 h and 0.23 ± 0.15 at 7d, Figure 3H).

CD206 positive cells were present in sham-operated mice (7.3 ± 0.9 cell/mm^2^). They could be observed 6 h after pMCAO (12.2 ± 6.2) and significantly increased progressively up to 24 h (23.6 ± 5.3 at 12 h and 40.0 ± 14.9 at 24 h). A significant number of CD206 positive cells was still present at 48 h (30.5 ± 10.5) and at 7d (32.7 ± 8.8, Figure 3I).

### Localization of M/M markers with respect to the lesion

**Figure 4:**
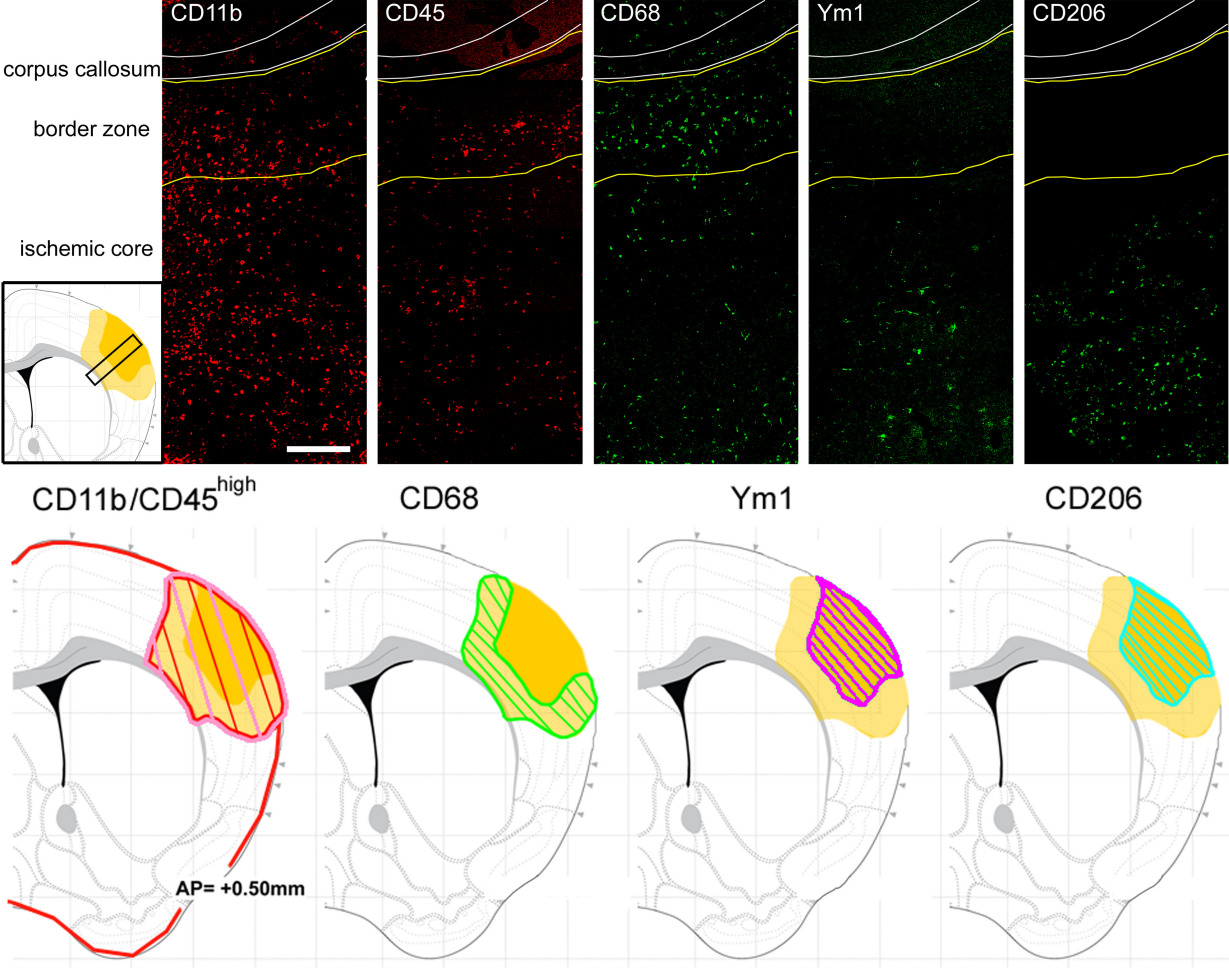
**Distribution of the selected M/M markers inside the ischemic lesion at 24 h from the injury**. Representative immunostaining micrographs show spatial distribution of M/M phenotype markers (CD11b, CD45, Ym1, CD206, CD68) into the ischemic area at 24 h after ischemia (upper panels). Bar = 250 μm. Drawings representing the immunostaining data (lower panels). Only immunoreactivity for CD11b could be observed outside the lesioned area in basal conditions (sham-operated mice). In the lesion, CD11b+ cells showing a ramified to globular morphology could be observed going from the border zone to the ischemic core (red). CD45 staining was present throughout the entire ischemic area (pink). Conversely CD68 appeared to be strongly concentrated in the border zone. Ym1 and CD206 were exclusively expressed in the ischemic core.

Twenty-four hours after pMCAO, the immunoreactivity for CD11b appeared to be evenly distributed in the ischemic area, being present both in the lesion border and in the ischemic core (Figure 4). At the same time CD45 staining showed a similar distribution being present throughout the entire ischemic area (Figure 4). CD45 cells visible at 10× magnification (Figure 4) did not reveal the presence of CD45^low ^cells (corresponding to ramified microglia) appearing in the CD11b staining microphotograph. Conversely CD68 appeared to be mainly concentrated in the border zone, with rare cells present in the ischemic core. Notably, at longer time points (7d) along with the great increase of its expression (Figure 3G), CD68 appears both in the border and in the core areas. Ym1 at 24 h after pMCAO appeared exclusively expressed in the ischemic core, similarly to CD206 (Figure 4). With the exception of CD68, all the markers considered showed a similar distribution at every time point (data not shown).

### Coexpression of M/M markers at 24 h and 7d after pMCAO

**Figure 5:**
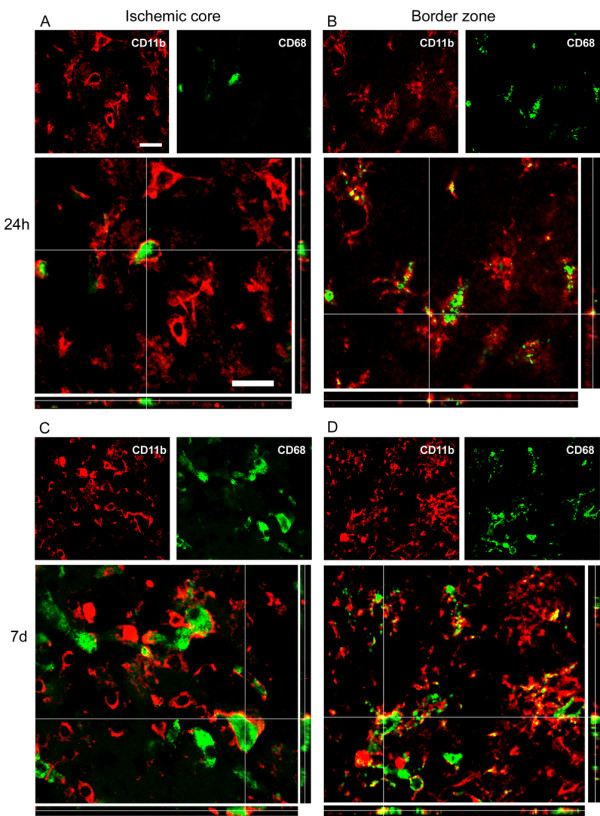
**Coexpression of CD11b (red) and CD68 (green) 24 h (A-B) and 7d (C-D) after pMCAO**. In the ischemic core at 24 h CD11b positive cells are prevalently globular and some of them are positive to CD68 (A). In the border zone (B) CD11b cells display rounded cell bodies and ramified processes positive to CD68. Globular CD11b cells in the lesioned area 7d after ischemia mostly express CD68 marker (C). A high number of CD11b cells displaying different morphology colabel with CD68 in the border zone (D). Data are representative of 3 independent experiments. Bars: 20 μm.

Twenty-four hours after ischemia CD68 was expressed in hypertrophic ameboid CD11b cells present in the ischemic core and in ramified microglia in the border zone where CD68 positive cells were mostly located (Figure 4 and 5A-B). A similar pattern of coexpression could be observed at 7d. At this time point the expression of CD68 was greatly increased both in globular CD11b+ cells in the ischemic core and in ramified CD11b cells laying in the border zone (Figure 4 and Figure 5C-D).

**Figure 6:**
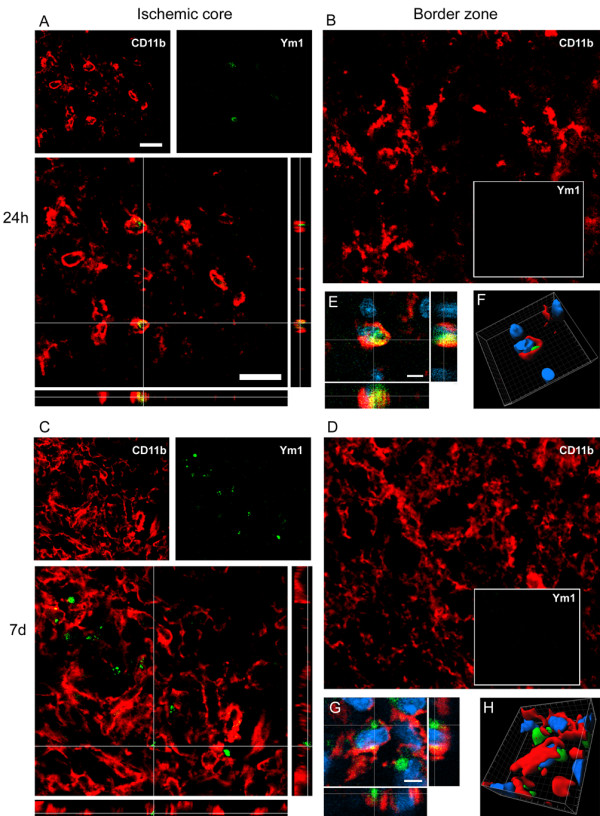
**Coexpression of CD11b (red) and Ym1 (green) at 24 h (A-B-E-F) and 7d (C-D-G-H) after pMCAO**. Ym1 positive cells co-label with globular CD11b postive cells at both time points (A, C). High magnifications (E-G) and 3D rendering (F-H) show colabeling of markers further highlighting the coexpression (blu = nuclei, bar: 5 μm). Consistent with the observation that no Ym1 cells are present in the border zone (Figure 4), no immunostaining for Ym1 at neither time points could be observed in this area (B, D). Data are representative of 3 independent experiments. Bars: 20 μm.

At 24 h after pMCAO Ym1 positive cells co-labeled with CD11b globular cells within the ischemic core, where they were exclusively located (Figure 4 and Figure 6A-B-E-F). Seven days after ischemia Ym1 and CD11b coexpression pattern was similar to that observed at 24 h (Figure 6C-D-G-H).

**Figure 7:**
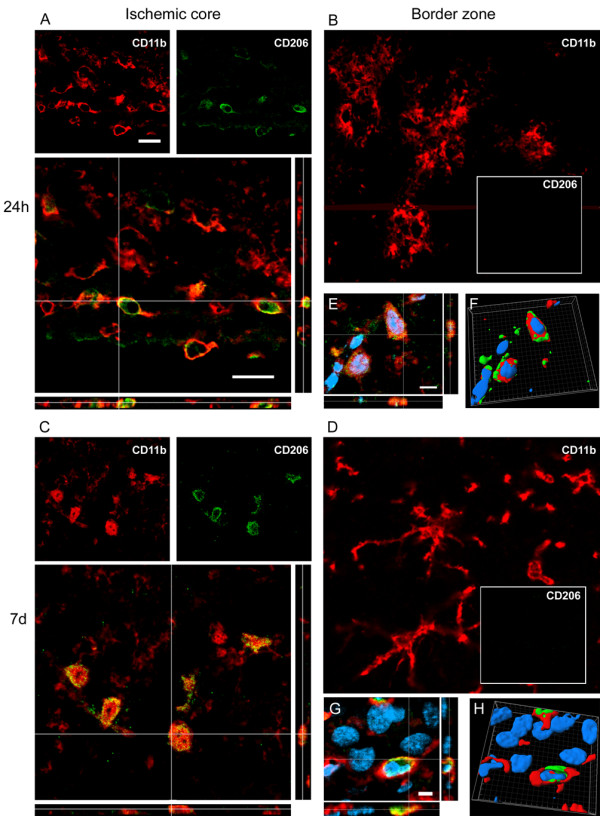
**Coexpression of CD11b (red) and CD206 (green) at 24 h (A-B-E-F) and 7d (C-D-G-H) after pMCAO**. At 24 h after ischemia some globular CD11b cells co-label with CD206 marker in the ischemic core (A). Seven days after ischemia CD11b cells are prevalently globular in the ischemic core and are highly positive to CD206 (C). High magnifications (E-G) and 3D rendering (F-H) show colabeling of markers further highlighting the coexpression (blu = nuclei, bar: 5 μm). Consistent with the observation that no CD206 cells are present in the border zone (Figure 4), no immunostaining for this marker at neither time points could be observed in this area (B, D). Data are representative of 3 independent experiments. Bars: 20 μm.

CD206 at 24 h was present exclusively in the ischemic core (Figure 4) where it colocalized with globular CD11b positive cells (Figure 7A-B-E-F). The same pattern of coexpression was observed at 7d (Figure 7C-D-G-H).

**Figure 8:**
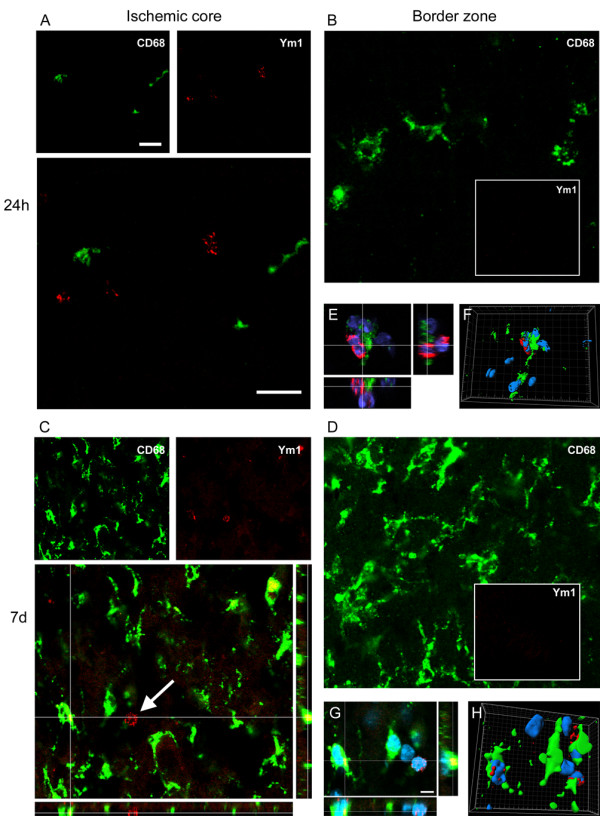
**Coexpression of CD68 (red) and Ym1 (green) at 24 h (A-B-E-F) and 7d (C-D-G-H) after pMCAO**. At 24 h CD68 positive cells found in the ischemic core do not co-localize with Ym1 positive cells (A). In high magnifications panels (E-F) the two markers appear to belong to different cells although in close contact (blu = nuclei). Seven days after ischemia, when CD68 immunoreactivity is greately increased, Ym1 appears to be expressed also, but not exclusively in CD68 positive cells (C). Note the presence of one Ym1 positive cells (arrow) that does not co-localize with CD68. High magnifications (G) and 3D rendering (H) show colabeling of markers further highlighting the coexpression (blu = nuclei). Consistent with the observation that no Ym1 cells are present in the border zone (Figure 4), no immunoreactivity for this marker could be observed at neither time points in that area (B, D). Data are representative of 3 independent experiments. Bars: 20 μm. High magnifications and 3D rendering bar: 5 μm.

At 24 h after pMCAO, the few CD68+ cells found in the ischemic core did not colocalize with Ym1+ cells that were present exclusively in this area (Figure 4 and Figure 8A-B). In the magnification of Figure 8E-F it is possible to observe that even when these markers appear closely related, they actually belong to distinct cells. At longer times (7d) Ym1 cells not colocalizing with CD68 are still present, however coexpression with CD68 can also be seen (Figure 8C-D-G-H).

**Figure 9:**
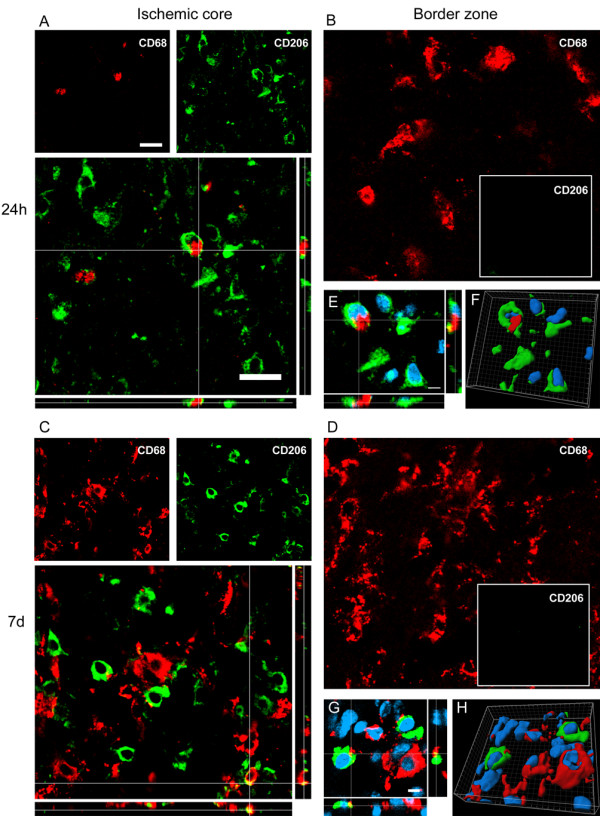
**Coexpression of CD68 (red) and CD206 (green) at 24 h (A-B-E-F) and 7d (C-D-G-H) after pMCAO**. At 24 h and 7d after ischemia, a minor part of CD68 positive cells found in the ischemic core colocalize with CD206 (A, C). High magnification (E-G) and 3D rendering (F-H) show both single- and double-positive cells in the ischemic core (blu = nuclei, bar: 5 μm). Consistent with the observation that no CD206 cells are present in the border zone (Figure 4), no immunoreactivity for this marker could be observed at neither time points in that area (B, D). Data are representative of 3 independent experiments. Bars: 20 μm.

A small fraction of CD206 positive cells show coexpression with CD68 at 24 h after ischemia in the ischemic core (Figure 9A-E-F). Similar situation is observed at 7d after pMCAO when a dramatic increase in CD68 positive cells is apparent in the ischemic core (Figure 9C-G-H). CD206 marker is not present in the border zone at both time points (Figure 9B-D).

**Figure 10:**
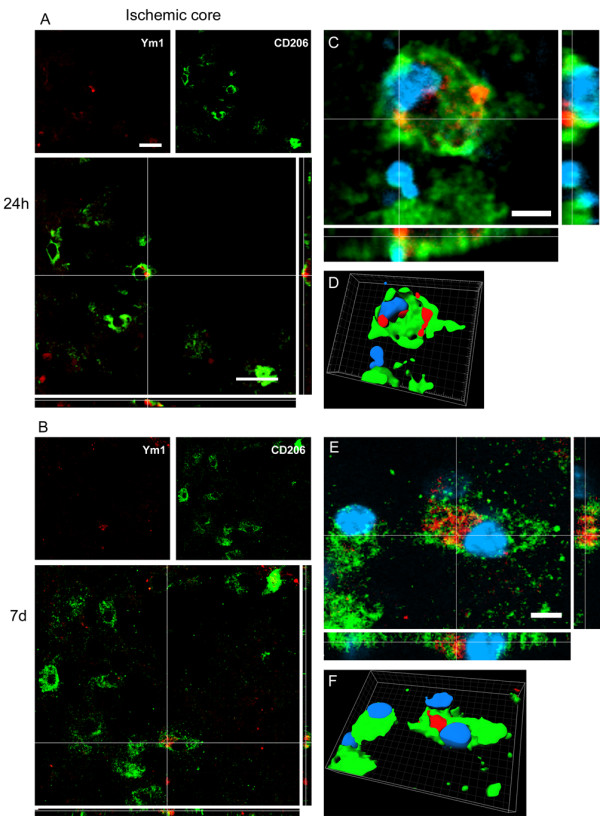
**Coexpression of Ym1 (red) and CD206 (green) at 24 h (A-C-D) and 7d (B-E-F) after pMCAO**. At 24 h and 7d after ischemia Ym1 positive cells co-label with CD206 positive cells in the ischemic core (A-B) Bars: 20 μm. High magnification (C-E) and 3D rendering (D-F) show coexpression of markers with the same cell nucleus (blu). Bar: 5 μm. Consistent with the observation that neither Ym1 cells nor CD206 cells are present in the border zone (Figure 4) no immunostaining for these markers could be observed at neither time points in this area (data not shown). Data are representative of 3 independent experiments.

Ym1 and CD206 appeared to be coexpressed in the ischemic core both at 24 h (Figure 10A-C-D) and 7d (Figure 10B-E-F) after pMCAO. None of the two markers was present in the border zone at both time points considered (Figure 4).

As expected all CD11b globular, CD68 globular, Ym1 and CD206 positive cells were all positive for CD45^high ^in both ischemic core and border zone (data not shown), being CD45 a common marker for immune cell populations [31,32].

**Figure 11:**
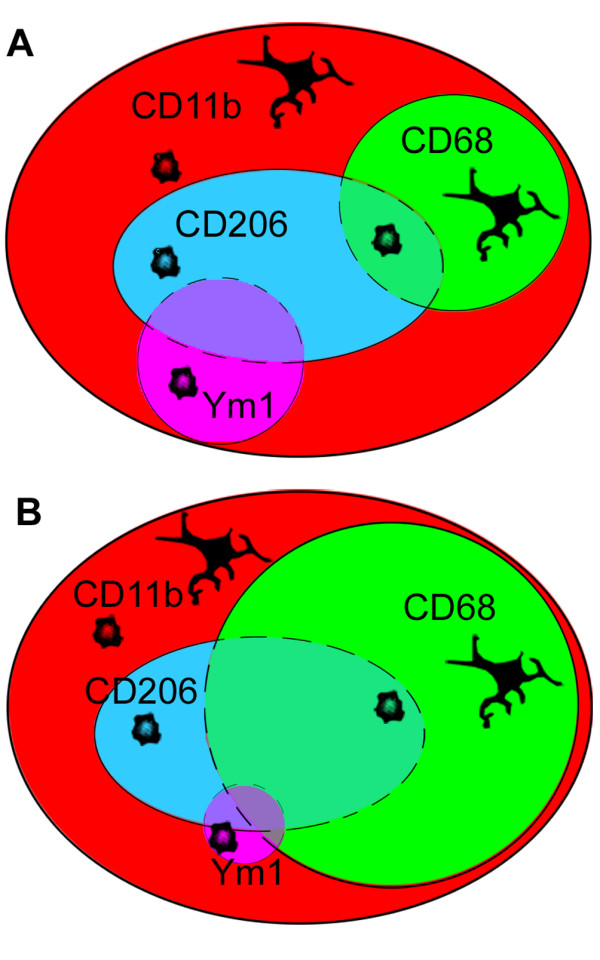
**Summary of M/M markers coexpression 24 h and 7d after the ischemic lesion**. At 24 h after injury (A) immunoreactivity for CD11, which is readly increased after ischemia, is expressed in ramified and globular cells. CD68 is present in a percentage of both globular and ramified CD11b+ cells. Ym1 and CD206 that are present mostly in the core of the lesion, are expressed by a fraction of globular CD11b+ cells and can be present on the same cells. A few cells coexpressing CD206 and CD68 can be found in the area between the core and the border zone where the two markers are mainly located respectively. At 7d after injury (B) Ym1 decreases while CD68 expression greatly increases and from the border zone where it was at earlier times it invades the ischemic core (see also data in Figure 3). A few CD68+ cells appear now to express Ym1.

A summary of M/M markers coexpression 24 h and 7d after the ischemic lesion is reported in Figure 11.

**Figure 12:**
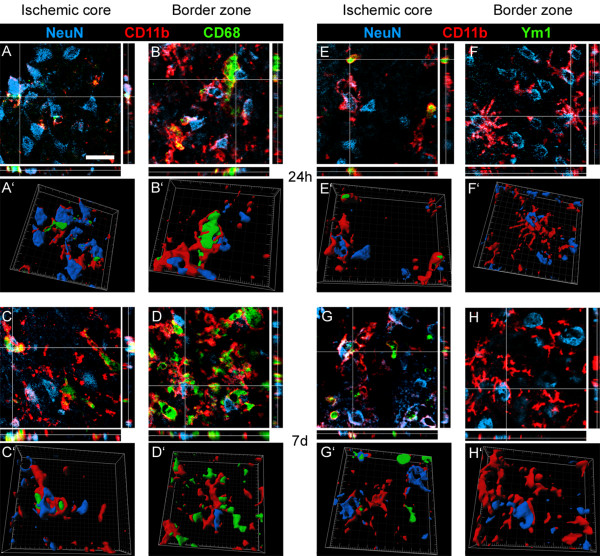
**Coexpression of CD11b (red) and NeuN (blue) with CD68 (green) at 24 h (A-A'-B-B') and 7d (C-C'-D-D') or with Ym1 at 24 h (E-E'-F-F') and 7d (G-G'-H-H') after pMCAO**. In the ischemic core CD11b/CD68 double positive cells envelop NeuN positive cells, possibly indicating phagocytosis of neurons (A; 3D rendering in A'). The same interaction was observed in the border zone (B-B'). At 7d after ischemia, when CD68 expression is enhanced (Figure 3) in both ischemic core and border zone, CD11b/CD68 double positive cells enwrap neurons, suggesting active phagocytosis also at this time point (C-C'-D-D'). At 24 h after pMCAO in the ischemic core, where Ym1 positive cells are exclusively located, CD11b/Ym1 double positive cells do not appear involved in a phagocytic interaction with neurons (NeuN positive cells, E; 3D rendering on E'). CD11b single positive cells in both ischemic core (E-E') and border zone (where Ym1 is not expressed, F-F') surround neurons. At 7d after ischemia part of CD11b/Ym1 double positive cells engage a phagocytic appearance by enveloping neurons in ischemic core (G-G') coherently with their partially CD68 positive phenotype at this time point (Figure 8). In the border zone at 7d (Ym1 is absent) CD11b single positive cells still enwrap neurons (H-H'). Data are representative of 3 independent experiments. Bar: 20 μm.

Lastly, to provide additional details on the functional status of M/M, we assessed their relationship with neurons (NeuN+). We analyzed CD11b/CD68 and CD11b/Ym1 double positive cells as these populations showed to increase at different time points, thus suggesting distinct functional states. CD11b stain of M/M membranes was chosen for documenting the morphology of M/M when contacting neurons. We found that neurons were often enwrapped by CD11b positive cells in both ischemic core and border zone at both 24 h and 7d (Figure 12). In most cases CD11b cells surrounding neurons were positive for CD68 at both zones (Figure 12A-B-C-D), suggesting an active phagocytosis. None of the CD11b/Ym1 double positive cells at 24 h appeared to be engaging a phagocytic interaction with neurons, being these cells never in contact with NeuN positive cells (Figure 12E). At 7d, a few CD11b/Ym1 double positive cells showed a phagocytic appearance enveloping neurons (Figure 12G), coherently with their partially CD68 positive phenotype at this time point (Figure 8). In the border zone, at both time points, Ym1 was not detectable and only single CD11b positive cells did envelop neurons (Figure 12F-H).

## Discussion

Our study shows that the ischemic lesion is accompanied by activation of specific M/M phenotype that presents distinctive spatial and temporal features. We have demonstrated that: 1) the ischemic lesion induces the expression of the selected M/M markers that develop over time, each with a specific pattern; 2) the selected markers are associated with globular or ramified CD11b morphology, 3) each marker has a given localization in the lesioned area with no apparent major changes during time, with the exception of CD68.

We have firstly determined the histopathological features of the lesion induced by pMCAO. From the analysis of the temporal evolution of the lesion it appears that the percentage of neuronal loss is somehow stable from 24 h up to 7d although the persistence of TUNEL-positive cells at this late point indicates that some cells may still be in degeneration at that late time. It should be noted that assessing the lesion volume by the paleness of the cresyl violet staining may lead to misleading conclusions since, as detailed below, invading inflammatory cells may contribute to the apparent reduction of the lesioned area at 7d. Actually the quantification of the CD11b and CD45^high ^immunoreactivity indicates that inflammatory cells rapidly increase in number and/or size early after the injury and at every time point considered.

M/M play a pivotal role in surveillance and response to altered CNS conditions [1,2,4]. An emerging concept is that, similarly to what happens for peripheral macrophages, these cells can exert different antithetic functions depending on environmental signals, acting as major players in the pro-inflammatory cytotoxic response, but also participating in the immunosuppressive and self-repair processes [6,33,34]. The phenotype markers considered in this study include classical markers of M/M activation (CD11b and CD45) and markers expressed by alternatively activated macrophages (CD68, Ym1 and CD206). Although evidence of M2 activation state in the brain has been reported in M/M in AD models [35,36], following global ischemia [37], in models of experimental autoimmune encephalomyelitis [38] or spinal cord injury [16,39], information on M2 marker expression, coexpression and temporal evolution in the injuried brain is lacking. We could observe that these phenotype markers are exclusively expressed by CD11b cells and that each of them shows distinct features in terms of time course of activation and localization in relation to the ischemic lesion.

CD11b is expressed on the surface of many leukocytes and is a widely used maker of M/M. It belongs to a family of cell surface receptors known as integrins. It is covalently bound to a beta 2 subunit to form integrin α_M_β_2 _(Mac-1, CD11b/CD18) which is implicated in diverse responses including cell-mediated killing, phagocytosis, chemotaxis and cellular activation. CD11b has the ability to recognize a wide series of ligands such as fibrinogen, iC3b fragment of the third complement component, ICAM-1, denaturated products, blood coagulation factor X [40]. Our data show that CD11b staining increases at early time points after ischemia, rapidly reaching a plateau of activation. Notably, CD11b positive cells display a different morphology in relation to the lesion, namely they are ramified in the border zone and ameboid in the ischemic core. Similarly to CD11b, also CD45^high ^cells increase rapidly after ischemia. These cells, display a rounded morphology and most probably correspond to recruited macrophages, neutrophils and lymphocytes [22,32,41]. This study does not specifically address the question of differentiating between invading macrophages and resident microglia. An important future direction will be to identify specific molecular/phenotypical markers for these two cell populations, since there is evidence that they may play a different role in the progression of brain injury [9,34,42].

CD68 or macrosialin is a member of the lysosomal/endosomal-associated membrane glycoprotein (LAMP) family and a member of the scavenger receptor family which recognizes a wide range of anionic macromolecules such as oxidatively modified lipoprotein, apoptotic cells and cell surface antigens of microorganisms. Its localization and predominance in phagocytic macrophages implicates CD68 in phagocytosis [43,44]. We observed that the early increase in CD68 immunoreactivity is concentrated in the border zone and expressed in ramified CD11b positive cells. At later time points a dramatic increase in CD68 expression appears both in the border zone and the ischemic core and is apparent in globular CD11b cells. At both time points and in both zones, CD11b/CD68 double positive cells appear to physically interact with neurons and show a phagocytic-like morphology characterized by neuron engulfment. The phagocytic activity of alternatively activated M/M is associated to clearance of cells debris, of damaged or dying cells and of infiltrating neutrophils thus resulting in the elimination of several potentially cytotoxic substances [4,18,21,45,46]. However the overall functional meaning of phagocytosis in acute brain injury is still an open question. Actually the protective effect of manipulations such as stem cell infusion may be associated with a decrease in CD68 expression [47].

Ym1 belongs to the lectin family and is constitutively expressed by liver, lung and bone marrow, consistently with the fact that these are the original sources of myeloid cells [48]. It is synthesized and secreted by activated macrophages during inflammation and exhibits a pH-dependent, specific activity towards GlcN oligomers and heparin. Ym1 may control leukocyte trafficking by competing with them for binding sites on local extracellular matrix, an action resulting in down-regulation of inflammation. Our findings show that, similarly to what reported in peripheral macrophages, Ym1 is activated transiently suggesting that it may be involved in the establishment of an inflammatory management control of the injured region [48]. Its expression is restricted to the ischemic core and it colocalizes with CD11b globular cells and with some CD68 cells at later times only. None of the CD11b/Ym1 double positive cells is associated with phagocytosis of neurons at 24 h, whilst at 7d some of them show a phagocytic appearance and envelop neurons, coherently with their partially CD68 positive phenotype at this time point. An increase in Ym1 expression has been associated to the beneficial effect of stem cell infusion in mice subjected to global ischemia [37], in line with a protective role in acute brain injury.

Another marker of alternatively activated macrophages is CD206 or mannose receptor [45,49]. This is an endocytic receptor that binds both microbial glycans and self glycoproteins carrying terminal mannose, fucose and N-acetylglucosamine by interaction with its carbohydrate recognition domains (CRDs). Its known function is related to recognition and endocytosis of the carbohydrate portion of antigens for processing and presentation [50]. Our results show that CD206 expression significantly increases over time and colocalizes with Ym1 positive cells and with a fraction of CD68 positive cells that increase at later time points.

Lastly, our data have been obtained in a model of permanent ischemia and may not be extended to an ischemia with reperfusion paradigm. Notably the present data and our previous results [22] indicate that the ratio of CD45^high^/CD45^low ^is dramatically different in these two conditions, being much higher after pMCAO. This may be due to either a higher number of infiltrating cells and/or a lower survival of resident cells, thus indicating that in transient ischemia the composition of the specific M/M populations in the lesioned area is different.

## Conclusions

In the ischemic lesion M/M express markers that show distinct temporal expression, distribution and association with a definite cell morphology suggesting that different M/M populations are acting at the site of injury according to well defined phenotype, time of activation and pattern of localization. Conceivably, at 24 h after insult, ramified and phagocytic M/M surround the lesion, possibly acting as a barrier against further expansion of the lesion, whilst globular M/M committed to a protective phenotype (*i.e*. expressing Ym1 and CD206) populate the ischemic core with the primary function of resolving inflammation and promoting wound healing. At later time points (7d) the phagocytic behavior of M/M becomes prevalent in the whole lesioned area with numerous globular phagocytic M/M invading the core territory. The observed switch towards the phagocytic phenotype is accompanied by the progressive reduction of the expression of the protective Ym1.

At 24 h protective Ym1 positive cells do not appear to be involved in neuron phagocytosis, differently from CD68 positive cells that show a close physical interaction with neurons. At 7d, when neuronal damage becomes irreversible in the core area, Ym1 positive cells decrease and start to show phagocytic behavior being partially co-localized with CD68. These cells now show the ability to envelop neurons in a phagocytic-like manner. Overall this effect suggests that endogenous protective mechanisms take place soon after injury (24 h-48 h) and last at least up to 7d when phagocytosis of neurons and debris removal are prevalent. Whether this effect is beneficial or detrimental cannot be clearly established. Phagocytosis (CD68 positive cells) can result in a protective function if properly balanced. In normal brain, phagocytic function of microglia have been suggested to support neurogenesis [51]. After an acute injury microglia are supposed to remove cellular parts, as well as whole cells [52], an action that might be necessary to remove irreversibly damaged cells to make space for new neuronal projections and fresh connections or newly generated neurons.

The different states of M/M in the ischemic lesion reflect the complexity of these cells and their ability to differentiate towards a multitude of phenotypes depending on the surrounding microenvironmental signals that can change over time. The inflammatory response that follows cerebral ischemia is regarded as a promising target for stroke therapy. The data presented in this study provide a basis for understanding this complex response and for developing strategies resulting in promotion of a protective inflammatory phenotype.

## List of abbreviations

BDNF: Brain Derived Neurotrophic Factor; CRDs: Carbohydrate Recognition Domains; DAB: 3, 3'-Diaminobenzidine; GDNF: Glial cell-Derived Neurotrophic Factor; ICAM: Inter-Cellular Adhesion Molecule; IGF-1: Insulin Growth factor-1; IL-1β: Interleukin-1β; LAMP: Lysosomal/endosomal-Associated Membrane Glycoprotein; M/M: Microglia/Macrophages; NO: Nitric Oxide; PBS: Phosphate Buffered Saline; pMCAo: permanent Middle Cerebral Artery occlusion; ROS: Reactive Oxygen Species; TNF: Tumor Necrosis Factor; TUNEL: Terminal Deoxynucleotidyl Transferase; dUTP Nick End Labeling.

## Competing interests

The authors declare that they have no competing interests.

## Authors' contributions

CP participated in the design of the experiments, carried out the experiments, acquired and interpreted the data, was involved in drafting the manuscript. SF carried out the experiments, acquired and interpreted the data, was involved in drafting the manuscript. MGDS participated in the design of the experiments, interpreted the data, was involved in drafting the manuscript. All authors read and approved the final manuscript.

## References

[B1] Davalos D Grutzendler J Yang G Kim JV Zuo Y Jung S Littman DR Dustin ML Gan WB ATP mediates rapid microglial response to local brain injury in vivo Nat Neurosci 2005 8 752 758 10.1038/nn1472 15895084

[B2] Yenari MA Kauppinen TM Swanson RA Microglial activation in stroke: therapeutic targets Neurotherapeutics 2010 7 378 391 10.1016/j.nurt.2010.07.005 20880502 PMC5084300

[B3] Iadecola C Anrather J The immunology of stroke: from mechanisms to translation Nat Med 2011 17 796 808 10.1038/nm.2399 21738161 PMC3137275

[B4] Jin R Yang G Li G Inflammatory mechanisms in ischemic stroke: role of inflammatory cells J Leukoc Biol 2010 87 779 789 10.1189/jlb.1109766 20130219 PMC2858674

[B5] Schilling M Besselmann M Muller M Strecker JK Ringelstein EB Kiefer R Predominant phagocytic activity of resident microglia over hematogenous macrophages following transient focal cerebral ischemia: an investigation using green fluorescent protein transgenic bone marrow chimeric mice Exp Neurol 2005 196 290 297 10.1016/j.expneurol.2005.08.004 16153641

[B6] Block ML Zecca L Hong JS Microglia-mediated neurotoxicity: uncovering the molecular mechanisms Nat Rev Neurosci 2007 8 57 69 10.1038/nrn2038 17180163

[B7] Hanisch UK Microglia as a source and target of cytokines Glia 2002 40 140 155 10.1002/glia.10161 12379902

[B8] Capone C Frigerio S Fumagalli S Gelati M Principato MC Storini C Montinaro M Kraftsik R De Curtis M Parati E De Simoni MG Neurosphere-derived cells exert a neuroprotective action by changing the ischemic microenvironment PLoS ONE 2007 2 e373 10.1371/journal.pone.0000373 17440609 PMC1847533

[B9] Lalancette-Hebert M Gowing G Simard A Weng YC Kriz J Selective ablation of proliferating microglial cells exacerbates ischemic injury in the brain J Neurosci 2007 27 2596 2605 10.1523/JNEUROSCI.5360-06.2007 17344397 PMC6672496

[B10] Neumann J Gunzer M Gutzeit HO Ullrich O Reymann KG Dinkel K Microglia provide neuroprotection after ischemia Faseb J 2006 20 714 716 16473887 10.1096/fj.05-4882fje

[B11] Nakajima K Yamamoto S Kohsaka S Kurihara T Neuronal stimulation leading to upregulation of glutamate transporter-1 (GLT-1) in rat microglia in vitro Neurosci Lett 2008 436 331 334 10.1016/j.neulet.2008.03.058 18406522

[B12] Stoll G Jander S The role of microglia and macrophages in the pathophysiology of the CNS Prog Neurobiol 1999 58 233 247 10.1016/S0301-0082(98)00083-5 10341362

[B13] Thored P Heldmann U Gomes-Leal W Gisler R Darsalia V Taneera J Nygren JM Jacobsen SE Ekdahl CT Kokaia Z Lindvall O Long-term accumulation of microglia with proneurogenic phenotype concomitant with persistent neurogenesis in adult subventricular zone after stroke Glia 2009 57 835 849 10.1002/glia.20810 19053043

[B14] Lu YZ Lin CH Cheng FC Hsueh CM Molecular mechanisms responsible for microglia-derived protection of Sprague-Dawley rat brain cells during in vitro ischemia Neurosci Lett 2005 373 159 164 10.1016/j.neulet.2004.10.004 15567573

[B15] Batchelor PE Liberatore GT Wong JY Porritt MJ Frerichs F Donnan GA Howells DW Activated macrophages and microglia induce dopaminergic sprouting in the injured striatum and express brain-derived neurotrophic factor and glial cell line-derived neurotrophic factor J Neurosci 1999 19 1708 1716 10024357 10.1523/JNEUROSCI.19-05-01708.1999 PMC6782182

[B16] David S Kroner A Repertoire of microglial and macrophage responses after spinal cord injury Nat Rev Neurosci 2011 12 388 399 10.1038/nrn3053 21673720

[B17] Porta C Rimoldi M Raes G Brys L Ghezzi P Di Liberto D Dieli F Ghisletti S Natoli G De Baetselier P Tolerance and M2 (alternative) macrophage polarization are related processes orchestrated by p50 nuclear factor kappaB Proc Natl Acad Sci USA 2009 106 14978 14983 10.1073/pnas.0809784106 19706447 PMC2736429

[B18] Michelucci A Heurtaux T Grandbarbe L Morga E Heuschling P Characterization of the microglial phenotype under specific pro-inflammatory and anti-inflammatory conditions: Effects of oligomeric and fibrillar amyloid-beta J Neuroimmunol 2009 210 3 12 10.1016/j.jneuroim.2009.02.003 19269040

[B19] Penninger JM Irie-Sasaki J Sasaki T Oliveira-dos-Santos AJ CD45: new jobs for an old acquaintance Nat Immunol 2001 2 389 396 11323691 10.1038/87687

[B20] Bhatia S Fei M Yarlagadda M Qi Z Akira S Saijo S Iwakura Y van Rooijen N Gibson GA St Croix CM Rapid host defense against Aspergillus fumigatus involves alveolar macrophages with a predominance of alternatively activated phenotype PLoS One 2011 6 e15943 10.1371/journal.pone.0015943 21246055 PMC3016416

[B21] Raes G Noel W Beschin A Brys L de Baetselier P Hassanzadeh GH FIZZ1 and Ym as tools to discriminate between differentially activated macrophages Dev Immunol 2002 9 151 159 10.1080/1044667031000137629 12892049 PMC2276098

[B22] Gesuete R Storini C Fantin A Stravalaci M Zanier ER Orsini F Vietsch H Mannesse ML Ziere B Gobbi M De Simoni MG Recombinant C1 inhibitor in brain ischemic injury Ann Neurol 2009 66 332 342 10.1002/ana.21740 19798727

[B23] Storini C Bergamaschini L Gesuete R Rossi E Maiocchi D De Simoni MG Selective inhibition of plasma kallikrein protects brain from reperfusion injury J Pharmacol Exp Ther 2006 318 849 854 10.1124/jpet.106.105064 16705080

[B24] De Simoni MG Storini C Barba M Catapano L Arabia AM Rossi E Bergamaschini L Neuroprotection by complement (C1) inhibitor in mouse transient brain ischemia J Cereb Blood Flow Met 2003 23 232 239 10.1097/01.WCB.0000046146.31247.A1 12571454

[B25] Swanson R Morton M Tsao-Wu G Savalos R Davidson C Sharp F A semiautomated method for measuring brain infarct volume J Cereb Blood Flow Metab 1990 10 290 293 10.1038/jcbfm.1990.47 1689322

[B26] Donnelly DJ Gensel JC Ankeny DP van Rooijen N Popovich PG An efficient and reproducible method for quantifying macrophages in different experimental models of central nervous system pathology J Neurosci Methods 2009 181 36 44 10.1016/j.jneumeth.2009.04.010 19393692 PMC2737682

[B27] Ortolano F Colombo A Zanier ER Sclip A Longhi L Perego C Stocchetti N Borsello T De Simoni MG c-Jun N-terminal kinase pathway activation in human and experimental cerebral contusion J Neuropathol Exp Neurol 2009 68 964 971 10.1097/NEN.0b013e3181b20670 19680147

[B28] Longhi L Gesuete R Perego C Ortolano F Sacchi N Villa P Stocchetti N De Simoni MG Long-lasting protection in brain trauma by endotoxin preconditioning J Cereb Blood Flow Metab 2011 10.1038/jcbfm.2011.42 PMC3185879 21468087

[B29] Santosh C Brennan D McCabe C Macrae IM Holmes WM Graham DI Gallagher L Condon B Hadley DM Muir KW Gsell W Potential use of oxygen as a metabolic biosensor in combination with T2*-weighted MRI to define the ischemic penumbra J Cereb Blood Flow Metab 2008 28 1742 1753 10.1038/jcbfm.2008.56 18545262 PMC3119432

[B30] Faul F Erdfelder E Lang AG Buchner A G*Power 3: a flexible statistical power analysis program for the social, behavioral, and biomedical sciences Behav Res Methods 2007 39 175 191 10.3758/BF03193146 17695343

[B31] Sedgwick JD Schwender S Imrich H Dorries R Butcher GW ter Meulen V Isolation and direct characterization of resident microglial cells from the normal and inflamed central nervous system Proc Natl Acad Sci USA 1991 88 7438 7442 10.1073/pnas.88.16.7438 1651506 PMC52311

[B32] Stein VM Baumgartner W Schroder S Zurbriggen A Vandevelde M Tipold A Differential expression of CD45 on canine microglial cells J Vet Med A Physiol Pathol Clin Med 2007 54 314 320 10.1111/j.1439-0442.2007.00926.x 17650152

[B33] Colton CA Heterogeneity of microglial activation in the innate immune response in the brain J Neuroimmune Pharmacol 2009 4 399 418 10.1007/s11481-009-9164-4 19655259 PMC2773116

[B34] Lambertsen KL Clausen BH Babcock AA Gregersen R Fenger C Nielsen HH Haugaard LS Wirenfeldt M Nielsen M Dagnaes-Hansen F Microglia protect neurons against ischemia by synthesis of tumor necrosis factor J Neurosci 2009 29 1319 1330 10.1523/JNEUROSCI.5505-08.2009 19193879 PMC6666095

[B35] Reed-Geaghan EG Reed QW Cramer PE Landreth GE Deletion of CD14 attenuates Alzheimer's disease pathology by influencing the brain's inflammatory milieu J Neurosci 2011 30 15369 15373 10.1523/JNEUROSCI.2637-10.2010 PMC2997622 21084593

[B36] Shin JW Lee JK Lee JE Min WK Schuchman EH Jin HK Bae JS Combined Effects of Hematopoietic Progenitor Cell Mobilization from Bone Marrow by Granulocyte Colony Stimulating Factor and AMD3100 and Chemotaxis into the Brain Using Stromal Cell-Derived Factor-1alpha in an Alzheimer's Disease Mouse Model Stem Cells 2011 29 1075 1089 10.1002/stem.659 21608078

[B37] Ohtaki H Ylostalo JH Foraker JE Robinson AP Reger RL Shioda S Prockop DJ Stem/progenitor cells from bone marrow decrease neuronal death in global ischemia by modulation of inflammatory/immune responses Proc Natl Acad Sci USA 2008 105 14638 14643 10.1073/pnas.0803670105 18794523 PMC2567180

[B38] Ponomarev ED Maresz K Tan Y Dittel BN CNS-derived interleukin-4 is essential for the regulation of autoimmune inflammation and induces a state of alternative activation in microglial cells J Neurosci 2007 27 10714 10721 10.1523/JNEUROSCI.1922-07.2007 17913905 PMC6672829

[B39] Kigerl KA Gensel JC Ankeny DP Alexander JK Donnelly DJ Popovich PG Identification of two distinct macrophage subsets with divergent effects causing either neurotoxicity or regeneration in the injured mouse spinal cord J Neurosci 2009 29 13435 13444 10.1523/JNEUROSCI.3257-09.2009 19864556 PMC2788152

[B40] Solovjov DA Pluskota E Plow EF Distinct roles for the alpha and beta subunits in the functions of integrin alphaMbeta2 J Biol Chem 2005 280 1336 1345 15485828 10.1074/jbc.M406968200

[B41] Gelderblom M Leypoldt F Steinbach K Behrens D Choe CU Siler DA Arumugam TV Orthey E Gerloff C Tolosa E Magnus T Temporal and spatial dynamics of cerebral immune cell accumulation in stroke Stroke 2009 40 1849 1857 10.1161/STROKEAHA.108.534503 19265055

[B42] Ajami B Bennett JL Krieger C McNagny KM Rossi FM Infiltrating monocytes trigger EAE progression, but do not contribute to the resident microglia pool Nat Neurosci 2011 14 1142 1149 10.1038/nn.2887 21804537

[B43] de Beer MC Zhao Z Webb NR van der Westhuyzen DR de Villiers WJ Lack of a direct role for macrosialin in oxidized LDL metabolism J Lipid Res 2003 44 674 685 10.1194/jlr.M200444-JLR200 12562841

[B44] Ramprasad MP Terpstra V Kondratenko N Quehenberger O Steinberg D Cell surface expression of mouse macrosialin and human CD68 and their role as macrophage receptors for oxidized low density lipoprotein Proc Natl Acad Sci USA 1996 93 14833 14838 10.1073/pnas.93.25.14833 8962141 PMC26222

[B45] Jayadev S Nesser NK Hopkins S Myers SJ Case A Lee RJ Seaburg LA Uo T Murphy SP Morrison RS Garden GA Transcription factor p53 influences microglial activation phenotype Glia 2011 59 1402 1413 10.1002/glia.21178 21598312 PMC3143257

[B46] Denes A Vidyasagar R Feng J Narvainen J McColl BW Kauppinen RA Allan SM Proliferating resident microglia after focal cerebral ischaemia in mice J Cereb Blood Flow Metab 2007 10.1038/sj.jcbfm.9600495 17440490

[B47] Zanier ER Montinaro M Vigano M Villa P Fumagalli S Pischiutta F Longhi L Leoni ML Rebulla P Stocchetti N Human umbilical cord blood mesenchymal stem cells protect mice brain after trauma Crit Care Med 2011 10.1097/CCM.0b013e31822629ba 21725237

[B48] Chang L Karin M Mammalian MAP kinase signalling cascades Nature 2001 410 37 40 10.1038/35065000 11242034

[B49] Porcheray F Viaud S Rimaniol AC Leone C Samah B Dereuddre-Bosquet N Dormont D Gras G Macrophage activation switching: an asset for the resolution of inflammation Clin Exp Immunol 2005 142 481 489 16297160 10.1111/j.1365-2249.2005.02934.x PMC1809537

[B50] Linehan JD Kolios G Valatas V Robertson DA Westwick J Immunomodulatory cytokines suppress epithelial nitric oxide production in inflammatory bowel disease by acting on mononuclear cells Free Radic Biol Med 2005 39 1560 1569 10.1016/j.freeradbiomed.2005.07.019 16298681

[B51] Sierra A Encinas JM Deudero JJ Chancey JH Enikolopov G Overstreet-Wadiche LS Tsirka SE Maletic-Savatic M Microglia shape adult hippocampal neurogenesis through apoptosis-coupled phagocytosis Cell Stem Cell 7 483 495 10.1016/j.stem.2010.08.014 PMC4008496 20887954

[B52] Kettenmann H Neuroscience: the brain's garbage men Nature 2007 446 987 989 10.1038/nature05713 17410127

